# Identification of tumor rejection antigens and the immunologic landscape of medulloblastoma

**DOI:** 10.1186/s13073-024-01363-y

**Published:** 2024-08-19

**Authors:** Changlin Yang, Vrunda Trivedi, Kyle Dyson, Tongjun Gu, Kate M. Candelario, Oleg Yegorov, Duane A. Mitchell

**Affiliations:** 1 UF Brain Tumor Immunotherapy Program, Preston A. Wells Center for Brain Tumor Therapy, Lillian S. Wells Department of Neurosurgery, University of Florida, 1333 Center Drive, BSB B1-118, Gainesville, FL 32610 USA; 2 Department of Biostatistics, University of Florida, Gainesville, FL USA

**Keywords:** Neoantigens, Tumor-associated antigens (TAAs), Medulloblastoma, Antigen landscape, Immunogenomics

## Abstract

**Background:**

The current standard of care treatments for medulloblastoma are insufficient as these do not take tumor heterogeneity into account. Newer, safer, patient-specific treatment approaches are required to treat high-risk medulloblastoma patients who are not cured by the standard therapies. Immunotherapy is a promising treatment modality that could be key to improving survival and avoiding morbidity. For an effective immune response, appropriate tumor antigens must be targeted. While medulloblastoma patients with subgroup-specific genetic substitutions have been previously reported, the immunogenicity of these genetic alterations remains unknown. The aim of this study is to identify potential tumor rejection antigens for the development of antigen-directed cellular therapies for medulloblastoma.

**Methods:**

We developed a cancer immunogenomics pipeline and performed a comprehensive analysis of medulloblastoma subgroup-specific transcription profiles (*n* = 170, 18 WNT, 46 SHH, 41 Group 3, and 65 Group 4 patient tumors) available through International Cancer Genome Consortium (ICGC) and European Genome-Phenome Archive (EGA). We performed in silico antigen prediction across a broad array of antigen classes including neoantigens, tumor-associated antigens (TAAs), and fusion proteins. Furthermore, we evaluated the antigen processing and presentation pathway in tumor cells and the immune infiltrating cell landscape using the latest computational deconvolution methods.

**Results:**

Medulloblastoma patients were found to express multiple private and shared immunogenic antigens. The proportion of predicted TAAs was higher than neoantigens and gene fusions for all molecular subgroups, except for sonic hedgehog (SHH), which had a higher neoantigen burden. Importantly, cancer-testis antigens, as well as previously unappreciated neurodevelopmental antigens, were found to be expressed by most patients across all medulloblastoma subgroups. Despite being immunologically cold, medulloblastoma subgroups were found to have distinct immune cell gene signatures.

**Conclusions:**

Using a custom antigen prediction pipeline, we identified potential tumor rejection antigens with important implications for the development of immunotherapy for medulloblastoma.

**Supplementary Information:**

The online version contains supplementary material available at 10.1186/s13073-024-01363-y.

## Background

Medulloblastoma is the most common malignant brain tumor in infants, children, and young adults, and it arises from progenitor cell populations present during cerebellum development [1–3]. Standard-of-care treatments including surgery, chemotherapy, and radiation therapy have greatly improved the survival of medulloblastoma patients but is responsible for life-long co-morbidities [4–8]. The disease remains fatal for about 25–30% of the patients with high-risk disease stratification and recurrence [4]. Advances in the field of cancer genomics and molecular profiling have provided significant insights into the development of the disease and its heterogeneity. Although considered a single disease, medulloblastoma encompasses four main molecular subgroups/subtypes—wingless (WNT), sonic hedgehog (SHH), Group3, and Group 4, each with unique transcription profiles, driver mutations, and prognosis [9–11].

WNT medulloblastoma is mainly dependent on mutations in the *CTNNB1* gene and the tumor suppressor gene *APC* which are responsible for driving the expression of the WNT signaling pathway and promoting tumor progression [12]. The SHH tumors have constitutively active SHH signaling pathways, with frequent mutations in the *PTCH1* gene and alterations in the *TP53* gene pathway [12]. Believed to be arising from a neural stem cell population, Group 3 tumors are associated with rare events of recurrent somatic mutations but have amplification of *MYCN*, *OTX2*, and *GFI1* and *GFI1B* genes, leading to tumor progression [12, 13]. Like Group 3 medulloblastoma, Group 4 has no defined driver pathway, and common somatic mutations are rare; however, amplification of *MYCN* and *OTX2* genes are frequent in Group 4 tumors [12]. In addition to these focal events, medulloblastoma tumors often have unstable genomes, with multiple chromosomal gains, losses, and fusions [12, 14]. It is important to note here that while the international consensus recognizes these four major subgroups of MB, there is further classification within the individual subgroups based on the molecular features such as gene expression and DNA methylation status, genetic alterations, age of the patients, clinical outcomes, and risk factors [15, 16]. In total, there are twelve additional subclassifications within the four major subgroups. Identification of these distinct disease entities, both in underlying biology and clinical characteristics, has influenced the design of refined preclinical studies and personalized treatment approaches.

The current personalized treatment strategies include drug-based targeted therapy to inhibit pathways involved in tumor progression such as BET-bromodomain inhibitor or HDAC and PI3K antagonists for MYC-driven Group 3 medulloblastoma or the inhibition of *SMO* and *CK2* in the SHH subgroup [17–21]. Beyond pharmacological agents, several promising immunotherapy modalities such as dendritic cell vaccines, adoptive cellular therapies, immune checkpoint inhibitors, and natural killer cell-based therapies are being evaluated for their efficacy preclinically and in early-phase clinical trials [22, 23]. In cancer, interactions between the T cell receptor on CD8 + T cells with MHC-I–peptide complex and CD4 + T cells with MHC-II–peptide complex are key to establishing tumor-specific T cell responses and effective immunotherapy. Peptide–MHC presentation is a necessary and highly selective step in the initiation of T cell activation, and peptides that cause T cell activation are termed antigens [24]. Cancer immunotherapies have traditionally targeted tumor-associated antigens (TAAs), which are self-antigens that are aberrantly overexpressed in cancer cells and as a result implied to play a role in tumor progression [24, 25]. Recently, attention has shifted to neoantigens and neoepitopes [26, 27]. Targeting an individual’s tumor-specific mutations is attractive because these neoepitopes are new to the immune system and not subject to host central tolerance. These mutations can be driver mutations driving the tumor progression or bystander mutations arising due to the genetic instability in the dividing tumor cells [28]. While targeting the driver mutations is more attractive, the tumor cells can be effectively killed by targeting any antigen, regardless of its oncogenic significance. Additionally, tumor-specific antigens arising from gene fusion events are also a class of neoantigens with the potential to elicit strong immunological responses [14]. The exploitation of the full repertoire of tumor antigens—that is, both unmutated antigens and neoantigens—may offer effective immunotherapy targets, especially for highly heterogeneous tumors with low mutation burdens like medulloblastoma.

Recent progress in the field of cancer immunogenomics has facilitated the search for tumor-specific antigens by applying comprehensive cancer genomics to tumor antigen discovery [29, 30]. We developed a pipeline called *O*pen *R*eading frame *A*ntigen a*N*alysis (O.R.A.N), which uses the gene expression data for identifying different classes of antigens. The current antigen prediction pipelines in the field predominantly identify neoantigens and more commonly MHC-I-associated antigens specific to CD8 + T cells [31–34]. The O.R.A.N pipeline further builds upon this platform to compute both MHC-I- and MHC-II-restricted antigens from a broad category of antigens including neoantigens, TAAs, and fusion proteins. In a recent study, we demonstrated the antigen prediction efficiency of the O.R.A.N pipeline in preclinical models of medulloblastoma and glioblastoma and the immunogenicity of the predicted antigens [35]. Additionally, we demonstrated the anti-tumor efficacy of antigen-directed immunotherapy targeting the predicted antigens [35].

Here, we set out to analyze the antigen landscape of human medulloblastoma subgroups using the O.R.A.N pipeline. We evaluated RNA-seq data from 170 medulloblastoma patient tumors made available through the International Cancer Genome Consortium (ICGC) and European Genome Phenome Archive (EGA) as well as an additional published dataset of 763 medulloblastoma gene expression profiles measured using microarray technology [12, 16]. While medulloblastoma tumors with subgroup-specific genetic substitutions have been previously reported, the immunogenicity of these genetic alterations and the extent of overlap of antigens expressed by the four subgroups remains unknown. Thus, the primary objective for this study was to determine (1) the number of predicted antigens for different classes of antigens such as neoantigens, TAAs, and fusion proteins for each medulloblastoma subgroup, (2) the recurrence of the same antigen in patients within a given medulloblastoma subgroup, and (3) the recurrence of the same antigen in patients across all four medulloblastoma subgroups. The information collected from this study is important to determine the scope of personalized or shared antigen-directed immunotherapeutic strategies for medulloblastoma tumors. To the best of our knowledge, this study is the first to present antigen prediction of TAAs and demonstrate that TAAs are correlated with survival in patients and serve as an attractive class of antigens in medulloblastoma, which has a low mutation burden. Additionally, we evaluated the antigen processing and presentation pathway in tumor cells and the immune infiltrating cell signature using the latest computational deconvolution methods to outline the immune landscape of the medulloblastoma tumors.

## Methods

### Data availability and quality control

We obtained patients’ RNA-seq data (*n* = 170; 18 WNT, 46 SHH, 41 Group 3, and 65 Group 4) (Additional file 1: Table S1) deposited at the EGA under accession number EGAS00001001953 (http://www.ebi.ac.uk/ega/) and associated mutational events with high biological significance from the ICGC Pan-Cancer Analysis of Whole Genomes (PCAWG) Dec. 2020 release [12, 36]. The patient demographic data is available in the supporting material for reference (Additional file 1: Table S1). Mutations including single nucleotide variation (SNV), insertions and deletions (indel), multi-nucleotide variation (MNV), and fusions were publicly available from https://dcc.icgc.org/releases/PCAWG. We also examined tumor SNVs other than single nucleotide polymorphisms. All RNA-seq data from this publication was downloaded and used for downstream epitope prediction analysis. RNA-seq read quality was assessed and universal adapters were trimmed using Trim Galore version 0.5.0 with a stringent threshold set to 5. An additional published dataset of 763 medulloblastoma gene expression profiles measured using microarray technology was downloaded from The National Center for Biotechnology’s Gene Expression Omnibus, available under accession GSE85218 [16]. For proteomics data, primary medulloblastoma patients’ tumor mass spectrometry data were acquired from Waszak et al. [37]. Only patients with available RNA-seq data were included in this analysis (*n* = 7).

### In silico haplotyping

Tumor RNA-seq reads were aligned to the International ImMunoGeneTics project HLA reference (IMGT/HLA, Release 3.32.0) using RazerS3, a component of SeqAn, an open-source C +  + library of efficient bioinformatics algorithms [38]. HLA class I haplotype of each patient was called with HLA Optitype (version 1.3.1) [39]. RNA-seq reads were also aligned to Human Genomic Reference Gencode version 23 (GRCh38.p3) using Bowtie2. HLA class II alleles were then extracted from the Bowtie 2 alignment data using the PHLAT algorithm (version 1.0) [40].

### Transcriptome expression profiling

Tumor RNA-seq data and normal tissue expression values obtained from the Toil Genotype-Tissue Expression Portal (GTEx) cohort were processed as mentioned. Briefly, quality-filtered tumor RNA-seq reads were aligned to the reference genome GRCh38.p3, and transcript expression was quantified with the RSEM (version 1.3.0) algorithm, as implemented by the University of California Santa Cruz Toil Recompute [41, 42]. Gene expression values were used as an additional filter for antigen prediction and for identifying TAAs and fusion proteins.

### Tumor neoantigen prediction

SNV and small indels were culled from the ICGC Pediatric Brain Tumors—Medulloblastoma & Pediatric Pilocytic Astrocytoma cohort—and Northcott et al. [12]. Mutation details were converted into Variant Call Format (VCF) 4.1 using a custom Linux script. VCF files from the two sources were annotated using Ensembl Variant Effect Predictor (VEP) GRCh37 version 91 [43], liftovered to Human CRCh38 VCF with Illumina Crossmap v0.3.9, and MHC-peptide binding affinity was estimated using the pVAC-Seq pipeline as implemented in pVACtools version 1.55 (NetMHCpan4.1 and NetMHCIIpan4.0) [44, 45]. Briefly, 8-12mer MHC-I-restricted peptides and 15mer MHC-II-restricted peptides were extracted from the annotated patient-specific variations. MHC-I-restricted epitopes were filtered in a stepwise fashion using the following parameters: predicted mutant peptide sequence binding affinity < 500 nM, RNA variant allele fraction (VAF) > 0.6, gene expression value of > 1 transcript per million (TPM). MHC-II-restricted peptides were filtered using the same parameters except that a binding affinity cutoff score of 1000 nM was used. The sequences of mutant epitopes were then screened by a non-redundant human peptides database. Only novel epitopes were considered putative antigens. In the following discussion, we will refer to neoantigens as immunogenic mutations harboring predicted epitopes. For mutations that harbor multiple epitopes, we still discuss it as a single neoantigen. The complete list of predicted immunogenic epitopes for individual mutations is provided in the supporting material (Additional file 2: Table S2).

### Gene fusions prediction

Gene fusions were called from RNA-seq alignments under STAR-Fusion [46] kickstart mode. Protein coding fusions were annotated with agfusion [47] and fed into the pVACtools pipeline. Fusion peptides’ binding affinity was determined in the same way as for neoantigen prediction. Potentially targetable and immunogenic gene fusions were then visualized with circlize package [48]. The complete list of predicted immunogenic epitopes for individual fusions is provided in the supporting material (Additional file 3: Table S3).

### Identification of tumor-associated antigens

Tumor RNA-seq data and normal tissue expression values were obtained and profiled as mentioned above. Normal tissues were divided into two groups: (1) normal tissues (29 organs or subregions, excluding EBV transformed, and cultured fibroblast cells, *n* = 9141) and (2) adult testis tissue (*n* = 170). We retrieved the mean plus two times the standard deviation (μ + 2σ) for all 29 organs/subregions to calculate the maximum expression of transcripts in normal tissue. To ensure that rare normal tissue expression outliers did not overly restrict our selection of candidate genes, we utilized the mean plus two standard deviations as our expression value and excluded genes with a value > 1. We identified tumor-associated genes (TAGs) as having TPM expression > 1 in tumor samples and TPM expression < 1 in normal tissues (using μ + 2σ values calculated as above). To ensure stringent identification of TAGs truly unique to the tumors, we considered any gene expression as the sum of expression of all transcripts (or isoforms) of that gene and applied the following threshold: gene TPM expression < 1 in the normal tissues and transcript TPM expression > 1 in tumors. When a gene expression in normal tissue is less than 1 TPM, it indicates that any corresponding transcript TPM value is also less than 1. Within each medulloblastoma tumor sample, a TAG was only included if its highest detectable transcript was also expressed at a TPM value above 1. Bedtools were then applied to tumor candidate TAGs by Samtools [49] and Bcftools [50] to extract consensus sequences. The human genomic reference was the same as above. We used all 9-12mer peptides derived from candidate TAGs for downstream MHC-I affinity prediction and 15mer for MHC-II affinity prediction. The prediction criteria was the same as mentioned for neoantigens and the TAGs harboring predicted immunogenic epitopes were called as TAAs. To ensure TAA epitope sequences were indeed tumor-specific, a peptide similarity filter was applied to all peptide-MHC selected epitopes which were screened against a customized human protein library and proteome database (Ensembl v82 peptides database) by Bash fgrep. This filter cleared all TAA epitopes that shared any homology with other protein-coding genes. The complete list of predicted immunogenic epitopes for individual TAA genes is provided on Dropbox due to size constraints (https://www.dropbox.com/scl/fi/oz2shf2glyiqx09qc6sy6/manuscript_0922.RData?rlkey=yne2c17aslieeu6ox5k696uek&dl=0) [51].

### Single-cell RNA-seq analysis

Primarily diagnosed medulloblastoma tumors' (*n* = 23) single-cell RNA-seq transcriptome data was acquired from Hovestadt et al. [52]. Data was reorganized with Seurat 4.0 [53]. Immunogenic TAAs (*n* = 99) previously selected from bulk RNA-seq cohort were mapped to the single-cell RNA-seq gene expression matrix. The top 3 frequently expressed genes based on single-cell RNA-seq data (*NEUROG1*, *IMPG2*, and *PIK3R3*) and pan-TAA (*n* = 99) were visualized at the single-cell level by subgroups.

### Proteomics analysis

The mass spectrometry raw data from ICGC matched medulloblastoma patients (*n *= 7, 1 SHH, e3 Group 3, and 4 Group 4) were obtained from the Pride database PXD016832 in the study conducted by Waszak et al. [37]. The raw data were converted into mzXML files using the Trans-Proteomic Pipeline. Subsequently, they were analyzed against antigens derived from the genomics analysis mentioned earlier, employing comet and x!Tandem algorithms with default settings. Results with a probability exceeding 0.7 were documented.

### Pathway enrichment analysis

All genes encoding for medulloblastoma antigens were functionally enriched for GO Terms, KEGG, REAC, and HPA database with the gprofiler [54] package selected using Bonferroni correction (BC) or Benjamin-Hochberg FDR algorithms (electronic annotations were excluded).

### Immune deconvolution

We downloaded 10 × single-cell RNA-seq data of 68,000 PBMCs sorted using flow cytometry into distinct immune cell populations [55]. Cells were selected when there were at least 1000 genes expressed. Genes selected for identifying immune cell populations spanned at least 3 samples. A single-cell reference matrix was constructed with CibersortX 1.0 [56]. Batch correction of this cross-platform analysis using the single-cell reference matrix above and 763 medulloblastoma patient’s microarray data was performed with S-Mode. Immune cell fractions were calculated by 100 permutations to receive an absolute score.

### Antigen-processing and presentation-related genes

Genes involved in antigen processing and presentation pathways (KEGG Map04612) were subset across medulloblastoma molecular subgroups and visualized by unsupervised hierarchical clustering with ComplexHeatmap [57]. Medulloblastoma subgroup-specific differentially expressed genes in Antigen Processing and Presentation pathway were visualized with Qiagen Ingenuity Pathway Analysis.

### Statistical analysis

Kruskal–Wallis test was performed for comparisons among medulloblastoma molecular subgroups. Wilcoxon signed-rank test was applied to pairwise comparisons between individual medulloblastoma subgroups. The *p* values (p.sig.signif) were corrected using Bonferroni correlation to control the type I error and were plotted using Alboukadel Kassambara’s rstatix and ggpubr packages. p.adj >  = 0.05 for “ns”, p.adj < 0.05 is “*,” p.adj < 0.01 is “**,” p.adj < 0.001 is “***,” and p.adj < 0.0001 is “****.” All data manipulations were performed using either Bash or R programming languages and run on the University of Florida high-performance computing cluster (HiPerGator).

### Code availability

Scripts for generating plots can be downloaded from GitHub (https://github.com/Mitchelllab/Medulloblastoma_Manuscript) [58].

## Results

### Development of the O.R.A.N pipeline

**Fig. 1:**
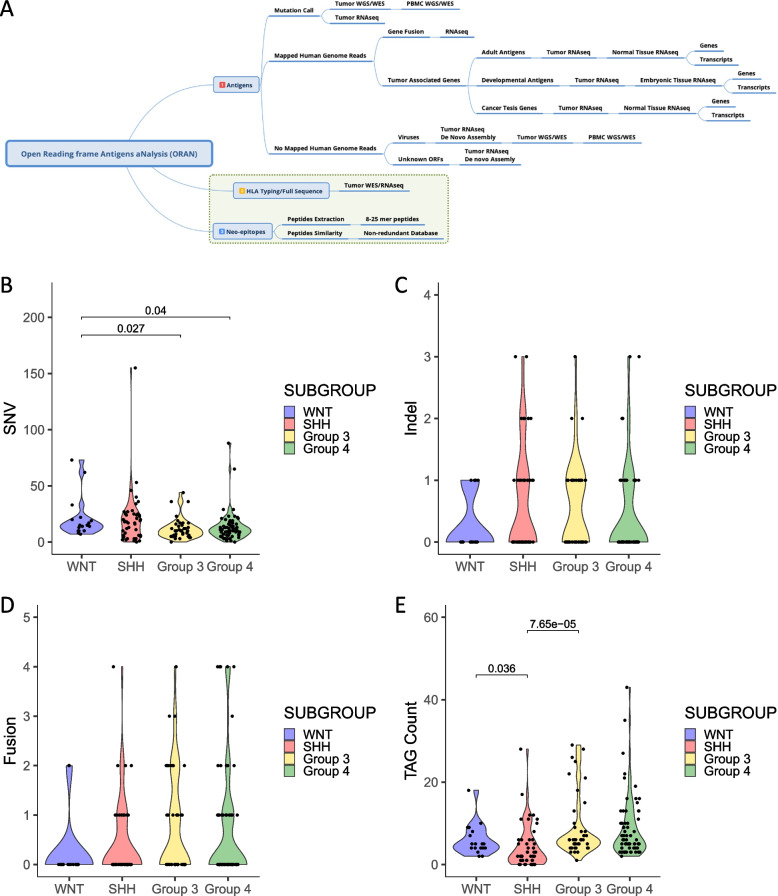
Potentially targetable genetic alterations and tumor-associated genes (TAGAs) in MB tumors. **A** The schematic for the Open Reading frame aNtigen Analysis (O.R.A.N) pipeline. **B** Single-nucleotide varations (SNVs) from human medulloblastoma (MB) tumors were plotted and grouped by MB molecular subgroups. **C** Intel count for small insertions and deletions were plotted and grouped by MB molecular subgroups. **D** Protein coding fusion counts detected for MB tumors were plotted and grouped by MB molecular subgroups. **E** Tumor-associated gene (TAG) counts were plotted and grouped by MB molecular subgroups. Patient tumor samples analyzed—*n* = 170; 18 WNT, 46 SHH, 41 Group 3, and 65 Group 4. Statistical analysis—Kruskal–Wallis and Wilcoxon tests, significance at *p* < 0.05

We obtained primarily diagnosed medulloblastoma (*n* = 170; 18 WNT, 46 SHH, 41 Group 3, and 65 Group 4) patient-specific mutations and transcriptome data from ICGC (PCAWG, Dec 2020) [12, 36]. Mutation burden included SNVs, indels, and MNVs obtained from publicly available database (https://dcc.icgc.org/releases/PCAWG) and used for downstream epitope prediction. The schematic of the pipeline is shown in Fig. 1A. However, we excluded MNVs from our study since most tumor's MNVs were found to be synonymous. We next determined the peptide binding prediction for MHC class I and II molecules utilizing the NetMHCpan4.1 and NetMHCIIpan4.0 tools, respectively [59]. These benchmark tools effectively integrate both binding affinity and eluted ligand data available from mass spectrometry, thereby providing state-of-the-art prediction of CD8 + and CD4 + T cell-associated epitope prediction. While most neoantigen and tumor-specific antigen prediction pipeline priorly published includes only MHC-I antigens, our pipeline has also incorporated MHC-II antigen prediction based on NetMHCIIpan4.0 network which covers all three MHC-II isotypes—HLA-DR, HLA-DQ, and HLA-DP and predict peptides of any length and for any MHC-II of known sequence [55]. This is particularly important because the CD4 + T cell helper cells have been shown to be crucial for sustained anti-tumor immunity [60]. Furthermore, we added a peptide similarity filter which eliminates all predicted epitopes which have a sequence homology to any protein-coding gene expressed in the normal tissue, thereby ensuring stringency in the antigen selection process to prevent any off-tumor targeting. Additionally, we derived protein coding gene fusions and TAGs using our antigen prediction pipeline O.R.A.N and performed immunogenic peptide prediction using the NetMHCpan4.1 and NetMHCIIpan4.0 tools mentioned above as well as the application of the downstream peptide similarity filter (Fig. 1A). Based on the above antigen prediction strategy, we predicted MB tumor-specific and tumor-associated antigens for the four major subtypes.

### Mutation burden and putative tumor-associated genes in medulloblastoma

We observed a low mutation burden across all four molecular subgroups (Fig. 1B–E). SNVs accounted for most of the variations while the numbers for indels and gene fusions per patient were quite similar. The average number of mutations in the WNT tumors was 20.9 (min = 7, max = 73), SHH was 19.7 (min = 0, max = 155), Group 3 was 11.8 (min = 0, max = 44), and Group 4 was 13.3 (min = 0, max = 88) (Fig. 1B). SNV mutations in Group 3 and Group 4 tumors were significantly lower than in WNT tumors (*p* = 0.027 and 0.04, respectively). On average, medulloblastoma patient tumors were found to have 15 mutations with the least being 0 mutations and the maximum being 155 mutations. On average, only 2 (min = 0, max = 3) indels and 2.5 (min = 0, max = 4) fusions were detected per patient tumor (Fig. 1C, D) with no statistically significant differences among the groups. Interestingly, only one patient’s tumor from the WNT subgroup expressed a protein-coding fusion (Additional file 4: Fig. S1).

To further identify potential tumor rejection antigens, we characterized TAGs. We acquired normal tissue gene and transcript expression from the TOIL-GTEx data portal and our strategy to characterize TAGs is mentioned in detail in the methods section. Overall, we found more TAGs in Group 3 and Group 4 than in the WNT and SHH tumors with the average number of TAGs in WNT being 6 (min = 2, max = 18), SHH being 4.3 (min = 0, max = 28), Group 3 being 9 (min = 1, max = 29), and Group 4 being 8.7 (min = 2, max = 43) (Fig. 1E). TAG count in Group 3 and WNT tumors was significantly higher than in SHH (*p* = 7.65e − 05 and 0.036, respectively). On average, medulloblastoma patient tumors were found to have 7 TAGs with the least being 0 TAGs and the maximum being 43 TAGs.

### Immunogenicity of tumor-specific mutations and neoantigen prediction

**Fig. 2:**
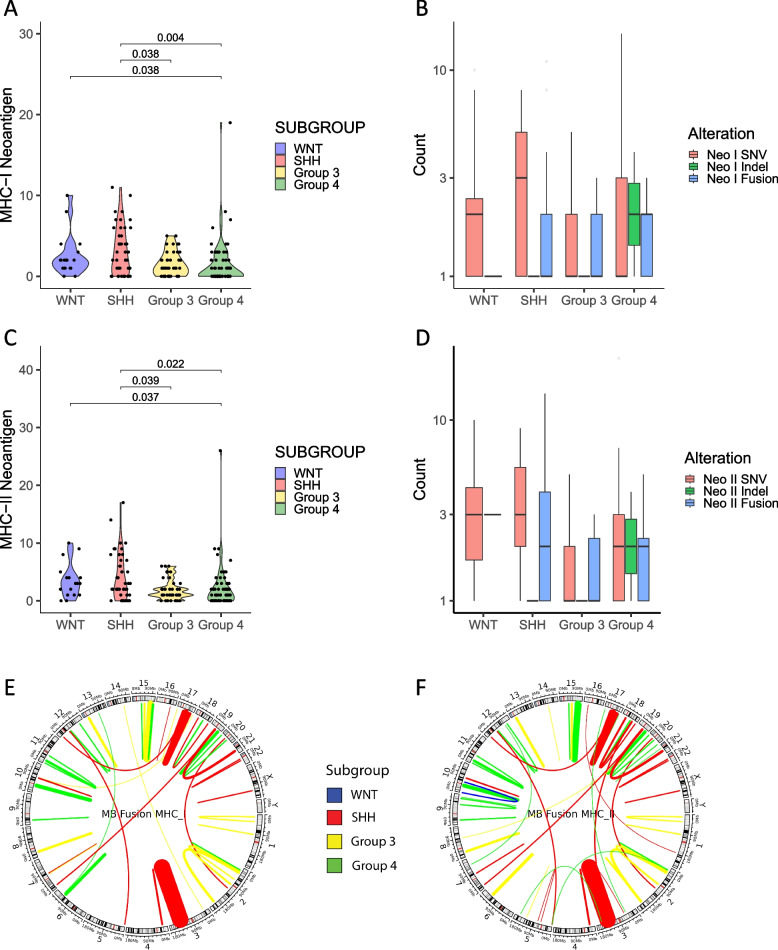
MHC class I- or class II-restricted neoantigens in MB tumors. **A** MHC-I-restricted neoantigens were plotted and grouped by MB molecular subgroups. **B** MHC-I-restricted neoantigens of MB tumors displayed by alteration types. **C** MHC-II-restricted neoantigens were plotted and grouped by MB molecular subgroups. **D** MHC-II-restricted neoantigens of MB tumors displayed by alteration types. **E** MHC-I- or **F** MHC-II-restricted fusion antigens’ expression based on chromosome distribution and MB subgroup displayed as chord plots. Patient tumor samples analyzed—*n* = 170; 18 WNT, 46 SHH, 41 Group 3, and 65 Group 4. Statistical analysis—Kruskal–Wallis and Wilcoxon tests, significance at *p* < 0.05

As HLAs are widely diverse, it is important to identify individual patients’ HLA haplotypes to make correct neoantigen predictions for each patient. Patient-specific HLA haplotypes were first predicted with high confidence using HLA haplotyping tools. Next, neoantigens were predicted based on the peptide-HLA binding affinity scores. As the mutation burden had suggested, the SNVs comprise the majority of the predicted neoantigens for each subgroup; however, not all identified SNVs were found to be immunogenic. Overall, the average number of MHC-I-restricted neoantigens found in WNT tumors was 2.6 (min = 0, max = 10), SHH was 3.2 (min = 0, max = 11), Group 3 was 1.6 (min = 0, max = 5), and Group 4 was 1.6 (min = 0, max = 19) (Fig. 2A, B). Similarly, the average number of MHC-II-restricted neoantigens found in WNT tumors was 3.5 (min = 0, max = 10), SHH was 4 (min = 0, max = 17), Group 3 was 2 (min = 0, max = 6), and Group 4 was 2.2 (min = 0, max = 26) (Fig. 2C, D). Group 4 predicted neoantigens were significantly lower than in WNT and SHH tumors for both MHC-I (*p* = 0.038 and 0.004, respectively) and MHC-II (*p* = 0.037 and 0.022, respectively) restricted antigens, while Group 3 predicted neoantigens were significantly lower than in SHH tumors for both MHC-I (*p* = 0.038) and MHC-II (*p* = 0.039) restricted antigens. We further examined if any subgroup was dominated by certain HLA allotypes which may create a bias and affect the number of immunogenic neoantigens predicted for that subgroup. We found that the most frequent HLA allotypes were associated with patient tumors from all four subtypes with some less frequent allotypes associating with a particular subgroup, demonstrating no major bias (Additional file 4: Fig. S2A).

The oncoprint of all 170 patient tumors is shown here to demonstrate the distribution of neoantigens (immunogenic mutations) based on individual medulloblastoma subgroups, the type of mutation (either SNVs or indels), and the gender, age, and vital status of the patients (Additional file 4: Fig. S2B). Over 95% of the neoantigen landscape was attributed to SNVs and several of the antigens were both MHC-I and MHC-II restricted. Additionally, the expression of neoantigens in individual subgroups was further evaluated (Table 1). All WNT (100%) tumors and most of the Group 3 (87.8%) and SHH (84.8%) tumors expressed at least one neoantigen, but only 64.6% of tumors from Group 4 expressed one neoantigen. Additionally, the number of immune-targetable neoantigens dropped quickly. Around 77.8% of WNT tumors and 56.5% of SHH tumors expressed three or more neoantigens as compared to the 29.3% of Group 3 tumors and 35.4% of Group 4 tumors. Overall, Group 3 and 4 tumors harbored fewer neoantigens compared to WNT and SHH. In all, there were a total of 390 immunogenic mutations out of 3018 mutations (SNVs and indels) (12.9%) identified from all patient tumors, demonstrating that not all identified mutations were immunogenic.

**Table 1:** Percentage of MB tumors by subgroup with at least one targetable neoantigen. MB tumors were analyzed for the expression of targetable neoantigens. Each tumor sample, organized by subgroup, was analyzed for the presence of the number of targetable neoantigens. The number of patient tumors with more than one, two, or three neoantigens was quantified and expressed as a percentage of the total number of tumors in their respective subtype. Patient tumor samples analyzed—*n* = 170; 18 WNT, 46 SHH, 41 Group 3, and 65 Group 4

Subgroup	One +	Two +	Three +
WNT	100%	88.9%	77.8%
SHH	84.8%	73.9%	56.5%
Group 3	87.8%	58.5%	29.3%
Group 4	64.6%	47.7%	35.4%

Interestingly, gene fusions were found to be highly immunogenic with most identified fusions in tumor samples (Additional file 4: Fig. S1) also having a high binding affinity for MHC-I- and II-restricted molecules as shown here with chord plots (Fig. 2E, F). In all, there were a total of 75 immunogenic fusions (69%) out of 108 fusions identified from all patient tumors, demonstrating that fusions are more immunogenic than neoantigens. Notably, previously reported fusions on chromosomes 17, 15, and 3 were also identified as potential immunogenic targets using our antigen prediction pipeline [9].

### Tumor-associated antigen prediction

**Fig. 3:**
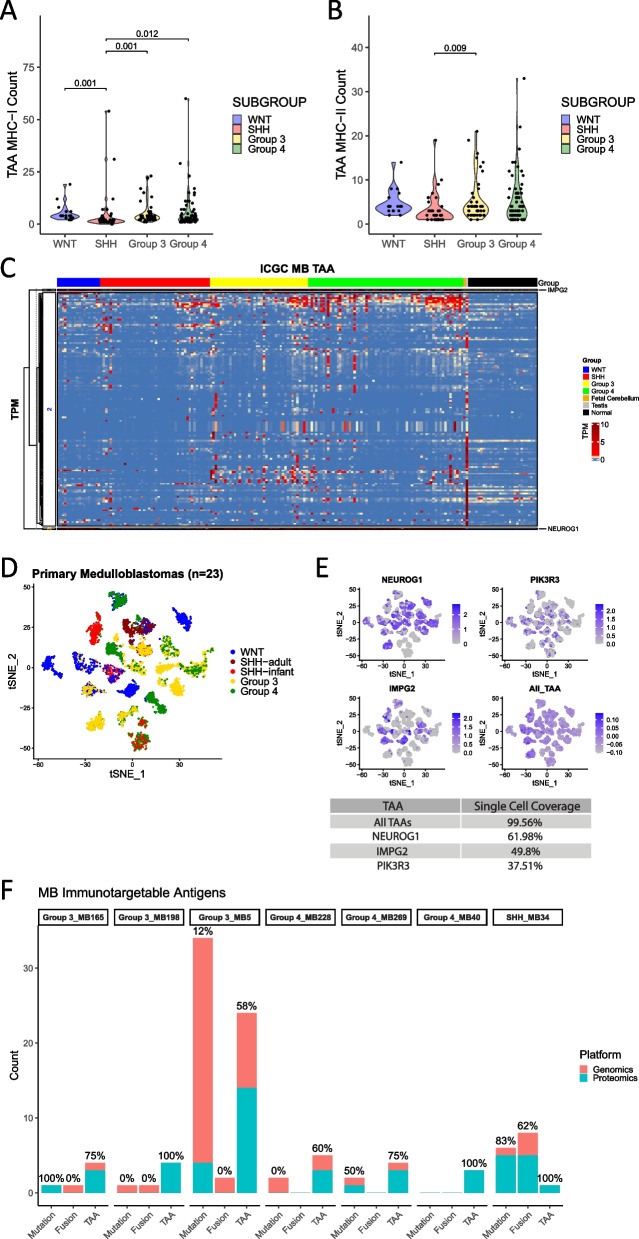
MHC class I or class II-restricted tumor-associated antigens (TAAs) in MB tumors and validation of antigen expression by proteomics. **A** MHC-I- and **B** MHC-II-restricted TAAs were plotted and grouped by each subgroup. Patient tumor samples analyzed—*n* = 170; 18 WNT, 46 SHH, 41 Group 3, and 65 Group 4. Statistical analysis—Kruskal–Wallis and Wilcoxon tests, significance at *p* < 0.05. **C** Unsupervised hierarchical cluster of immunogenic TAAs (*n* = 99) across MB tumors (*n* = 170). Maximum gene values of the following normal tissues- fetal cerebellum (*n* = 5), adult testis (*n* = 170), and 29 sub-organs or regions of adults were appended. Genes with TPM expression < 1 were from yellow to blue, and >1 showed from light red to dark red. **D** tSNE plot overlooking MB tumors based on single-cell RNA-seq data (*n* = 23). **E** Projection of top 3 abundant TAAs derived from single-cell RNA-seq data and 99 pan-TAAs identified from patient tumors using bulk-RNA seq. Blue dots are cells expressing the selected TAAs and gray dots represent no expression. **F** The number of immunogenic epitopes found from patient-matched genomics and proteomics data. TAAs from all three subgroups were found to have the most concordance between the two platforms

TAAs represent a class of antigens that are highly expressed in tumors but exhibit low or negligible expression in normal tissues. However, through sophisticated analyses, TAAs have been robustly linked to cancer-testis antigens (CTA) or antigens related to the cell-of-origin (considered as developmental antigens), which are fundamental in the initiation and development of tumors [61, 62]. We utilized the O.R.A.N pipeline to predict immunogenic TAAs, from the previously identified TAGs in each subgroup. Overall, Groups 3 and 4 tumors expressed the greatest numbers of TAAs for both MHC-I and MHC-II-restricted antigens (Fig. 3A, B). The average number of TAAs with MHC-I-restricted antigens in WNT tumors was 5.3 (min = 2, max = 19), SHH was 3.5 (min = 0, max = 54), Group 3 was 5.3 (min = 1, max = 23), and Group 4 was 5.6 (min = 0, max = 60). SHH tumors were found to have a significantly lower number of immunogenic TAAs than all the other groups (*p* = 0.001 for WNT, *p* = 0.001 for Group 3, and *p* = 0.012 for Group 4). Similarly, the average number of TAAs with MHC-II-restricted antigens in WNT tumors was 4.8 (min = 2, max = 14), SHH was 2.6 (min = 0, max = 19), Group 3 was 5.7 (min = 1, max = 21), and Group 4 was 5.3 (min = 0, max = 33). SHH tumors were found to have a significantly lower number of immunogenic TAAs than in Group 3 (*p* = 0.009). Additionally, we evaluated the expression of immunogenic TAAs in individual subgroups (Table 2). All WNT (100%) tumors and most of the Group 3 (97.6%) and Group 4 (98.5%) tumors expressed at least one TAA, but only 60.9% of tumors from SHH had one immunogenic TAA. Around 88.9% of WNT, 80.5% of Group 3, and 67.7% of Group 4 tumors expressed three or more TAAs as compared to the 34.8% of SHH tumors. In all, there were a total of 143 predicted immunogenic TAAs (86%) out of 166 TAGs identified from all tumors, showing that tumor-associated genes are highly immunogenic, provided the larger amount of sequence to evaluate antigens from.

**Table 2:** Percentage of MB tumors by subgroup with at least one targetable TAA. MB tumors were analyzed for the expression of targetable TAAs. Each tumor sample, organized by subgroup, was analyzed for the presence of the number of targetable TAAs. The number of patient tumors with more than one, two, or three TAAs was quantified and expressed as a percentage of the total number of tumors in their respective subtype. Patient tumor samples analyzed—*n* = 170; 18 WNT, 46 SHH, 41 Group 3, and 65 Group 4

Subgroup	One +	Two +	Three +
WNT	100%	100%	88.9%
SHH	60.9%	43.5%	34.8%
Group 3	97.6%	92.7%	80.5%
Group 4	98.5%	81.5%	67.7%

We applied an unsupervised clustering of TAAs within medulloblastoma along with fetal cerebellum tissue expression (*n* = 5), adult testis tissue expression (*n* = 170), and the maximum values of gene expression (using the μ + 2σ values calculated) in normal tissue (29 organs/subregions) (see the “Methods” section) (Fig. 3C). The unsupervised clustering of TAAs with fetal cerebellum tissue and adult testis tissue expression was performed to characterize TAAs which are shared between the tumor and these tissues as developmental antigens or CTA respectively. TAAs got split into three clusters by gene expression, two of which were defined by only a single widely expressed gene. Cluster 1 consisted of gene *IMPG2*, which was highly expressed in WNT, Group 3, and Group 4 tumors. Cluster 2 consisted of immunogenic TAAs that were also upregulated in the testis and were therefore considered as CTA. However, not all TAAs in cluster 2 were identified as CTAs. Cluster 3 consisted of a single oncofetal antigen, *NEUROG1*, which was shared among WNT, Group 3, and Group 4 tumors and overexpressed in the fetal cerebellum. *NEUROG1* is a known signature gene within the RL-svz (rhombic lip subventricular zone), playing a foundational role in the formation of Group 4 medulloblastoma (MB) [63].

We further studied TAA distribution by re-analysis of single-cell RNA-seq data (*n* = 23 patients) acquired from Hovestadt et al. [52] (Fig. 3D, E). The top 3 TAAs most frequently expressed by cells were oncofetal antigen *NEUROG1* (cluster 3 of the heatmap in Fig. 3C), *IMPG2* (cluster 1), and *PIK3R3*. *NEUROG1* was expressed in most tumors across all subgroups but not all the cells in each patient’s tumor expressed *NEUROG1*. *NEUROG1* was expressed in 61.98% of cells, while *IMPG2* and *PIK3R3* were expressed in 49.8% and 37.51%, respectively. We further mapped 99 TAAs identified from bulk RNA-seq data to their expression on the single-cell level, and we found that nearly all medulloblastoma tumors and their cells (99.56% of cells) expressed the predicted TAAs. Additionally, mass spectral data of matched patients’ tumors (one SHH patient, three Group 3 patients, and three Group 4 patients) was used for validation of mutations and TAAs on the protein level (Fig. 3F). We matched the predicted epitope sequences for neoantigens (SNVs, indels, and gene fusions) and TAAs to the peptides identified by mass spectrometry. Despite the dataset being limited to only 7 MB patients, there was a significant overlap between the antigens predicted through the O.R.A.N pipeline. Mutations (SNVs and indels) were found to be expressed at the protein level in 4 out of 7 patients’ tumors, while only one patient’s tumor from SHH expressed fusion epitopes. TAAs were robustly expressed at both gene and protein levels in all 7 patient tumors. Of note, the TAA prediction had the highest concordance with the proteomic dataset, with greater than 50% overlap for all patients and a 100% overlap shown for 3 out of the 7 patient samples evaluated. This further highlights the importance of TAAs and adds to the significance of our pipeline.

### Recurring antigens within medulloblastoma subgroups

**Fig. 4:**
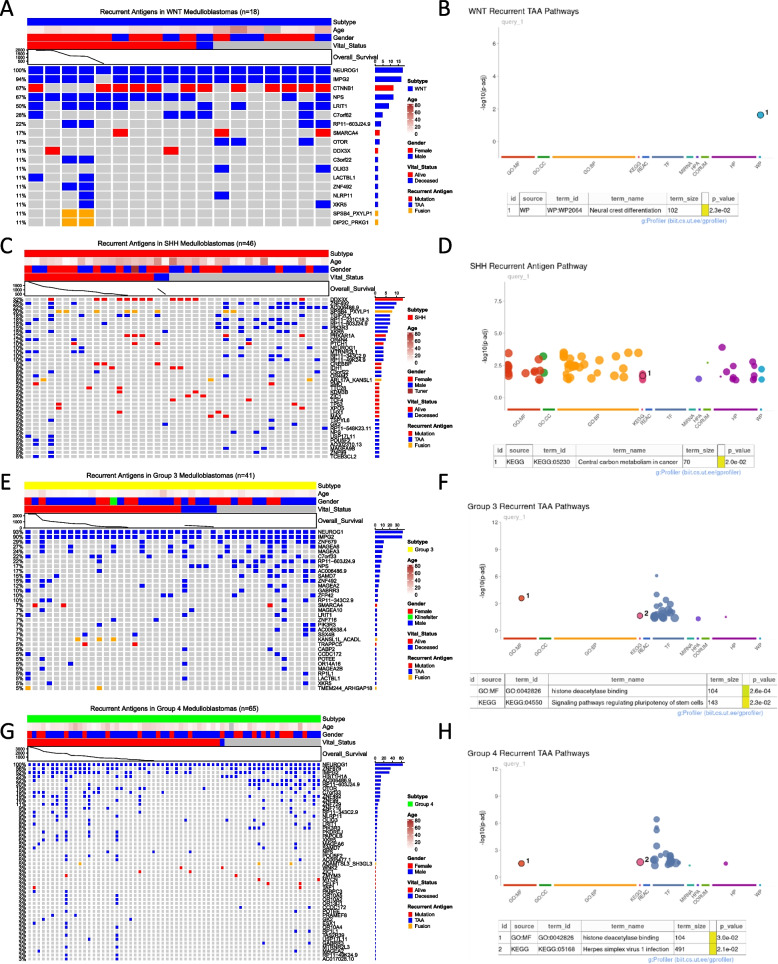
Subgroup-specific recurring antigen landscape and pathway enrichment analysis. **A**, **C**, **E**, and **G** Oncoprint illustrating recurring antigens in tumors. The figure displays antigens observed in at least two patients for the **A** WNT (*n* = 18), **C** SHH (*n* = 46), **E** Group 3 (*n* = 41), and **G** Group 4 (*n* = 65) subgroups. Immunogenic mutations (depicted in red), fusions (in orange), and TAA (in blue) are organized from the most frequently recurring antigens to the least, presented row-wise. Patient arrangement within molecular subgroups is based on survival events, with the columns indicating patient status as alive (depicted in red), deceased (in blue), and not available (in gray). This layout enables tracking the correlation between patients’ clinical data and their recurring antigens. **B**, **D**, **F**, and **H** Pathway enrichment analysis for recurring antigens within each subgroup is depicted—**B** WNT, **D** SHH, **F** Group 3, and **H** Group 4. Recurring antigens from molecular subgroups underwent over-representation analysis using the g:profiler R package. Fisher’s exact test was employed to filter significantly upregulated pathways (adjusted *p* value < 0.05). Enriched pathways are presented as dot plots, with pathway families on the *x*-axis and transformed *p* values on the *y*-axis

To determine which are the most commonly expressed antigens in medulloblastoma and how frequently these antigens recur across patient tumors, we looked at the recurring antigen landscape within each molecular subgroup. We defined any antigen expressed in at least two patient tumors as a recurring antigen. We identified 19 recurring antigens in WNT tumors (*n* = 18) with TAAs being the most commonly expressed antigens (14 out of 19 antigens). The frequently recurring TAAs included *NEUROG1*, *IMPG2*, *NPS*, *LRIT1*, and *C7orf62*. Notably, *CTNNB1*, one of the key mutations involved in driving the WNT tumors, was the only highly expressed neoantigen in these tumors (Fig. 4A). Pathway enrichment analysis for the recurring TAAs showed neural crest differentiation as the most interesting pathway enriched in WNT tumors (Fig. 4B). We identified 40 recurring antigens in SHH tumors (*n* = 46) of which 14 were mutations, 24 were TAAs, and 2 were arising from fusion proteins (Fig. 4C). Top neoantigen targets for SHH included *DDX3X*, *PRKAR1A*, and *PTCH1* which are genes commonly involved in tumor progression [12]. Top TAA targets for SHH included *ZNF492*, *AC006486.9*, *TGIF2LX*, *PIK3R3*, and *XKR5* among others. Nearly 20% of SHH patients had a fusion antigen arising from the *SPSB4-PXYLP1* fusion protein. Interestingly, there was a cohort/cluster of patient tumors that expressed fewer antigens than others and mainly lacked recurring TAA expression. The most notable pathway enriched for the recurring antigens in SHH was Central Carbon Metabolism in Cancer (Fig. 4D).

The recurring antigen landscape for Group 3 tumors (*n* = 41) showed 33 antigens out of which only 4 were neoantigens derived either from mutation or gene fusion (Fig. 4E). The rest 29 antigens were all expressed by TAAs and *NEUROG1* and *IMPG2* were expressed by over 90% tumors, making them the most suitable antigen targets for Group 3 medulloblastoma. Other notable TAA targets included the MAGE family of proteins such as *MAGEA6*, *MAGEA3*, *MAGEA2*, and *MAGEA10* and *ZNF679*, *ZNF492*, *C7orf33*, and *PIK3R3* among others. The 2 mutation-derived neoantigens included *SMARCA4* expressed in 3 patient tumors and *TRAPPC5* expressed in 2 patient tumors out of the 41 analyzed. Pathway enrichment analysis for the recurring TAAs showed that histone deacetylase binding and regulating pluripotency of stem cells pathways were enriched in Group 3 tumors (Fig. 4F). Like Group 3, Group 4 tumors (*n* = 65) also highly expressed TAAs, which comprised 43 out of the 50 identified antigens (Fig. 4G). *NEUROG1* was expressed by 100% of Group 4 tumors, making it a promising candidate for immunotherapy. Additional frequently expressed TAAs included *ZNF679*, *IMPG2*, *HIST1H1A*, *ZNF492*, and *OTOR* among others. Neoantigens were shared amongst very few patients, with only 2 out of the 78 patient tumors expressing *WNK2*, *MYCN*, *ZIC1*, *ZMYM3*, *CELF1*, and *TAF1* neoantigens. Only one fusion neoantigen derived from *ADAMTSL3-SH3GL3* was expressed in these patients. And like Group 3, the histone deacetylase binding pathway was enriched in Group 4 TAA antigens along with the pathway involved in herpes simplex virus 1 infection (Fig. 4H). Overall, most of the recurring antigens in each subgroup were TAAs with only SHH tumors having a substantial number of recurring neoantigens (Additional file 4: Fig. S3A).

When asked if there is a correlation between antigen load and the overall survival (OS) or progression-free survival (PFS) in patients, we observed a moderate correlation between MHC-I- and MHC-II-restricted SNVs and PFS in Group 4 medulloblastoma patients, with correlation coefficients of 0.64 and 0.52, respectively (Additional file 4: Fig. S3B–C, and Additional file 5: Table S4). Furthermore, robust correlations were identified between MHC-I- and II-restricted TAAs and OS in Group 3 medulloblastoma patients, demonstrating correlation coefficients of 0.79 and 0.77, respectively (Additional file 4: Fig. S3D–E). Moreover, these MHC-I and II TAAs displayed strong correlations with PFS among Group 3 medulloblastoma patients with correlation coefficients of 0.97 and 0.97, respectively (Additional file 4: Fig. S3F–G).

### Shared antigens by medulloblastoma subgroups

**Fig. 5:**
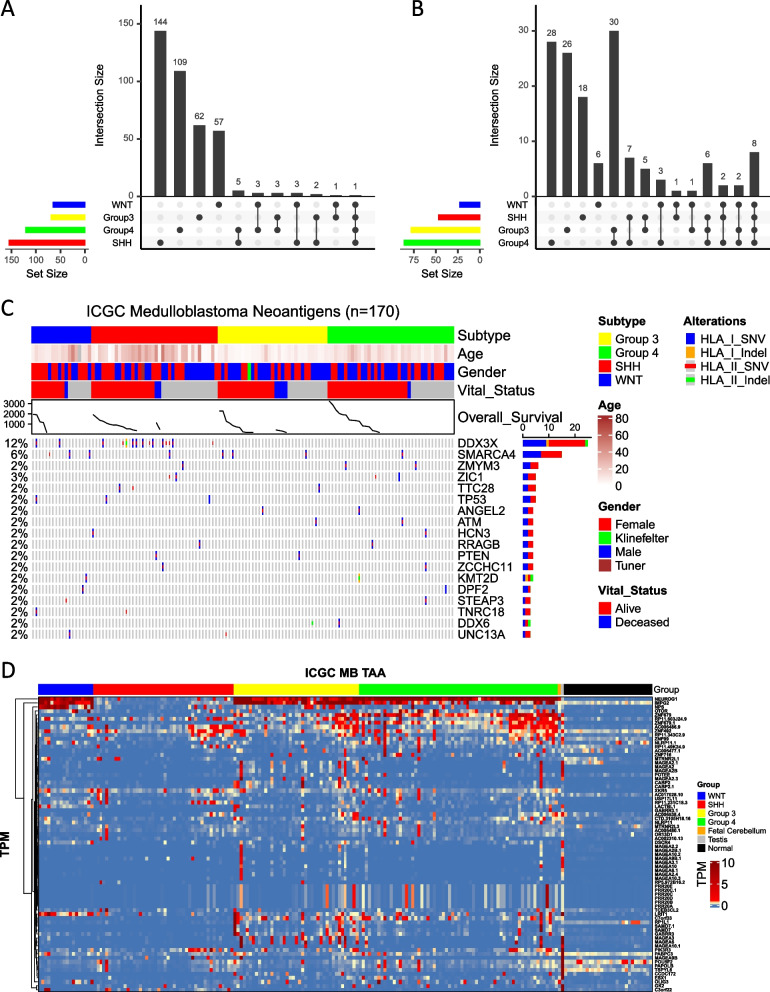
Analysis of shared antigens across all the four medulloblastoma subgroups. **A** Upset plot demonstrating private and shared predicted neoantigens across the 4 subgroups. **B** Upset plot demonstrating private and shared predicted TAAs across the 4 subgroups. **C** Oncoprint of shared neoantigens spanning at least two patient tumors across all the four subgroups. **D** Unsupervised hierarchical clustering of shared TAAs spanning at least two patient tumors across all four subgroups. Maximum values of the following normal tissues fetal cerebellum (*n* = 5), adult testis (*n* = 170), and 29 sub-organs or regions of adults were appended. Genes with TPM expression < 1 were from yellow to blue, and over 1 showed from light red to dark red. The bar across the top of the figure—blue = WNT, red = SHH, yellow = Group 3, and green = Group 4

After evaluating the antigens recurring within each molecular subgroup, the antigens that were shared by medulloblastoma patients (*n* = 170) across all subgroups were evaluated to determine the feasibility of developing a single immunotherapy approach that may serve all. We defined any antigen expressed in at least two patients’ tumors as a shared antigen. There was a total of 18 neoantigens that were shared by at least 2 medulloblastoma subgroups but only one neoantigen was shared by all (Fig. 5A). *DDX3X* was the most frequently shared neoantigen (12% of patients); however, it was expressed by only WNT and SHH tumors, while *SMARCA4* was the second most frequently occurring neoantigen (6% of patients) but was expressed by tumors across all 4 subgroups (Fig. 5C). *DDX3X* expression on WNT and SHH has priorly been known and is implicated in the survival, growth, and malignant potential of the tumor cells [64]. *SMARCA4* has been implicated in the genetic and epigenetic network that plays an important role in medulloblastoma tumor development and growth [65, 66]. Additional commonly shared neoantigens included *ZMYM3*, *ZIC1*, *TP53*, *ANGEL2*, *PTEN*, and *KMT2D* among others.

Interestingly, a lot more TAAs were found to be shared among subgroups, with 47 TAAs shared among at least two subgroups, 10 TAAs among 3 subgroups, and 8 TAAs among all 4 subgroups (Fig. 5B). Group 3 and Group 4 tumors had the greatest number of shared TAAs (30 total). The TAAs shared among all subgroups included *NEUROG1*, *AC006486.9 (novel protein)*, *NPS*, *OTOR*, *ZNF679*, *RP11.603J24.9 (AC034102.2*, *novel protein)*, *ZNF492*, and *LRIT1* (Fig. 5D).

### Antigen presentation

**Fig. 6:**
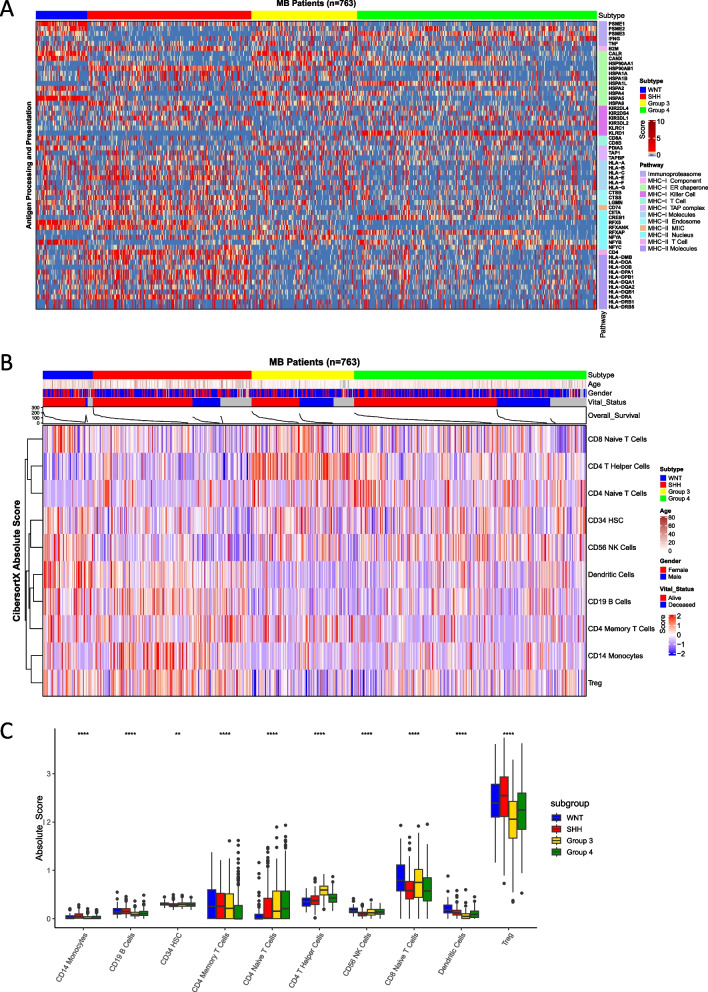
Antigen presentation and processing pathway enrichment analysis and immune cell landscape of medulloblastoma tumors. **A** Unsupervised hierarchical clustering of the genes involved in the antigen presentation pathway organized by MB subgroups. Expression of genes is shown on a scale from blue to dark red, with blue showing downregulation and red showing upregulation of genes. The bar across the top of the figure—blue = WNT, red = SHH, yellow = Group 3, and green = Group 4. **B** Digital cytometry of MB patient tumors organized by MB subgroup. Gene signatures were derived from a single-cell RNA-seq dataset of purified PBMCs and then applied to MB patients’ tumor microarray data (*n* = 763). The bar across the top of the figure—blue = WNT, red = SHH, yellow = Group 3, and green = Group 4. **C** Immune infiltration scores of each immune population from the previous deconvolution

To evaluate whether putative antigens can be effectively presented by medulloblastoma tumors, we analyzed the expression of genes involved in the antigen processing and presentation pathway in these tumors using a published dataset of 763 medulloblastoma gene expression profiles measured by microarray technology [16, 29]. Antigen processing and presentation pathways were significantly downregulated in Group 3 medulloblastoma tumors (yellow bar) compared to the remaining three subgroups (Fig. 6A). Unsupervised hierarchical clustering of genes demonstrated that Group 3 medulloblastoma tumors turned off antigen presentation pathways by downregulating MHC-II expression (Fig. 6A, Additional file 4: Fig. S4). There were no significant changes in antigen processing and presentation pathways among other subgroups; however, Group 4 tumors (green bar) showed downregulation of genes involved in MHC-I expression as observed from the unsupervised clustering of genes. Downregulation of MHC-I as a potential immune escape mechanism has also been demonstrated previously in medulloblastoma [67].

### Medulloblastoma immunological landscape

Previous work in the field has identified distinct immune cell landscape of the medulloblastoma subgroups using gene expression and single-cell RNA sequencing data [68, 69]. Here, we employed digital cytometry to deconvolute bulk medulloblastoma transcriptome data to reveal the immunological landscape of medulloblastoma [49]. Unsupervised hierarchical cluster indicated immune infiltrates clustered by medulloblastoma molecular subgroups (Fig. 6B). More myeloid-derived immune cells such as dendritic cells and monocytes were upregulated in WNT and SHH tumors along with B cells, (Fig. 6B, C) as compared to Group 3 and Group 4. This is in line with previously reported work where myeloid cell populations were significantly more abundant in SHH compared to Group 3 and Group 4 tumors as uncovered using single-cell RNA-sequencing data [69]. Regulatory T cells (Treg) were highly enriched in all medulloblastoma subgroups compared to the other immune cell populations but showed greater enrichment in WNT and SHH tumors as compared to Group 3 and Group 4. In contrast, Group 3 tumors showed higher infiltration of lymphoid-derived CD4 helper T cells compared to the other subgroups. Group 4 tumors showed no higher infiltration of a particular immune cell population compared to others; however, NK cells were slightly more enriched in Group 4 versus the other subgroups. CD8 memory T cells were not detected in any subgroup and were therefore excluded from the figures. We further evaluated if there is any correlation between the immunological scores and antigen prediction and found that TAAs, but not neoantigens, demonstrated a robust correlation with immunological scores (Additional file 6: Table S5). This may be due to the higher number of HLA-I and HLA-II epitopes predicted for TAGs compared to mutations, provided the longer length of sequences to predict antigens from.

## Discussion

Current therapies for the treatment of medulloblastoma impose life-long co-morbidities on developing children and highlight the need for safer and patient-specific personalized treatment approaches [6–8]. The precision medicine approach can be tailored to take advantage of the patient’s tumor’s genetic alterations for the development of immunotherapies such as personalized vaccines and adoptive T cell therapy [35]. Vaccine therapy is very promising as it can introduce a diverse array of tumor antigens and activate systemic tumor antigen-specific T-cell reactivity to enhance anti-tumor immune response [35, 70]. However, tumors such as medulloblastoma typically have a low mutation burden and heterogenous expression of antigens, which leads to immune resistance and subsequent escape. To address this intra-tumoral antigen heterogeneity, strategies to expand T cell populations with specificity for multiple antigens is being developed. One important consideration for antigen-directed immunotherapy is the identification of immunogenic antigens with epitopes that have strong binding affinity to patients’ HLA molecule. To this end, we developed an antigen prediction algorithm called O.R.A.N which predicts immunogenic antigens across a broad array of antigen classes such as neoantigens, TAAs, and fusion antigens.

Out of 3018 non-synonymous mutations identified in the medulloblastoma patient tumors, only 390 immunogenic mutations were projected, demonstrating poor immunogenicity of mutations. Across all 170 patients, we found that 79.4% of patients harbored at least one neoantigen, which indicated that most but not all patients may benefit from neoantigen-based immunotherapies. However, only 44% of patients express 3 or more than 3 neoantigens, indicating that neoantigen-based therapies may not be sufficient for medulloblastoma patients. Interestingly, TAAs showed a good occurrence rate in patients’ tumors. Out of the 166 TAGs identified, 143 were predicted to be immunogenic, demonstrating that TAGs are highly immunogenic. Additionally, 88.2% of patients harbored at least one TAA while 64% of the patients express more than 3 TAAs. The TAA prediction also showed a higher concordance with the proteome data as compared to neoantigens. Our single-cell RNA-seq data analysis showed that while a limited number of TAA antigens may not target all malignant cells, targeting pan-TAAs may target all the cells and solve the challenge of intra-tumoral heterogeneity. Additionally, our findings strongly suggest that TAAs hold promise as a valuable biomarker for medulloblastoma patients. The robust correlations observed between MHC-I and MHC-II TAAs with both overall survival and progression-free survival in the worst outcome Group 3 medulloblastoma patients emphasize the potential of TAAs as a biomarker for assessing disease prognosis in this specific subset of patients. Overall, TAAs may be a good supplementary of immunogenic targets to neoantigens.

Most medulloblastoma tumors in this study were predicted to have multiple private antigens which may facilitate the development of personalized pan-antigen reactive immunotherapy. Additionally, several frequently recurring and shared antigens were identified within the four subgroups, highlighting the scope of a common immunotherapy approach for broader recipient of patients. Particularly, immunotherapies targeting neoantigens from oncogenic driver mutations such as *CTNNB1*, *DDX3X*, and *SMARCA4* and TAAs which have possible implications in tumor progression such as *NEUROG1* and *PIK3R3* are attractive for a better outcome in patients. Multiple preclinical studies and on-going clinical trials including ReMATCH (NCT01326104) and ACTION (NCT03334305), among others, currently underway at our center, have utilized tumor RNA as a source of antigens. Our preclinical data have demonstrated the successful generation of anti-tumor T cells, leading to prolonged survival benefit in tumor-bearing host [35, 71]. Other studies have similarly demonstrated anti-tumor response with vaccines or antigen-specific T cells targeting medulloblastoma tumors or other pediatric high-grade gliomas in preclinical models [14, 72]. Furthermore, the tumor antigen vaccine can synergize effectively with other immune therapies, amplifying their efficacy [35].

Previously, Wells et al. had proposed a list of questions that can be used as a resource by the scientific community to benchmark and improve antigen prediction pipelines [73]. We compared our pipeline to the list of features mentioned in this study and found that O.R.A.N pipeline meticulously addressed 39 features out of the 49-question survey, ensuring comprehensive scrutiny of the antigen prediction process. The future iterations of the pipeline will focus on including additional features such as TCR binding. To further expand the tumor antigen repertoire and thus increase the number of targetable antigens, we aim to update O.R.A.N to identify and predict the immunogenicity of other classes of antigens such as splice variants and viral epitopes. The antigens predicted using the O.R.A.N pipeline can be leveraged into developing antigen-specific immunotherapies for medulloblastoma and can lead to favorable outcomes by addressing the tumor heterogeneity and immune escape challenges [35].

## Conclusions

Using our antigen prediction pipeline O.R.A.N, we show that medulloblastoma patients express multiple private and shared immunogenic antigens which can be leveraged as potential tumor rejection antigens. The antigen landscape of medulloblastoma tumors highlights the need for personalized antigen-directed immunotherapy to target private antigens, while also presenting an opportunity to target frequently occurring shared oncogenic drivers for a more universal immunotherapy approach. In conclusion, our study has important implications for the development of antigen-directed immunotherapy for medulloblastoma.

### Supplementary Information

Additional file 1: This file includes the demographic information of the 170 medulloblastoma patients whose RNAseq data was used in this study.

Additional file 2: This file includes the information about all epitopes identified from neoantigens for each patient.

Additional file 3: This file includes the information about all epitopes identified from fusion proteins for each patient.

Additional file 4: This file includes additional figures from S1 through S4 discussed in this study.

Additional file 5: This file includes the correlation of antigen type with overall survival and with progression-free survival.

Additional file 6: This file includes the correlation between antigen type and immune cell subsets.

## Data Availability

The RNA-seq data (*n* = 170) is deposited at the EGA under accession number EGAS00001001953 (https://ega-archive.org/studies/EGAS00001001953), and the associated mutational events are publicly available from the ICGC Dec. 2020 release (https://dcc.icgc.org/releases/PCAWG) [12, 36]. The microarray (*n* = 768) and proteomics data (*n* = 8) are obtained from NCBI GEO under GSE85218 (https://www.ncbi.nlm.nih.gov/geo/query/acc.cgi?acc=GSE85218) and Pride database under PXD016832 (https://proteomecentral.proteomexchange.org/cgi/GetDataset?ID=PXD016832), respectively [16, 37]. The raw antigen counts can be obtained from Dropbox (https://www.dropbox.com/scl/fi/oz2shf2glyiqx09qc6sy6/manuscript_0922.RData?rlkey=yne2c17aslieeu6ox5k696uek&dl=0) [51]. Scripts and data for generating plots in this paper can be downloaded from GitHub (https://github.com/Mitchelllab/Medulloblastoma_Manuscript) [58]. Codes for O.R.A.N antigen prediction pipeline are currently under the licensing process at the University of Florida but can be made available upon reasonable request.
