# Supplementary material for: Identification of tumor rejection antigens and the immunologic landscape of medulloblastoma
Source: Genome Med. 2024 Aug 19;16:102. doi: 10.1186/s13073-024-01363-y (PMC11331754; doi:10.1186/s13073-024-01363-y)
Supplement: Supplementary file 4 — Additional file 4: This file includes additional figures from S1 through S4 discussed in this study. [file 13073_2024_1363_MOESM4_ESM.docx]

**Identification of tumor rejection antigens and the immunologic landscape of medulloblastoma**

Changlin Yang*, Vrunda Trivedi*, Kyle Dyson, Tongjun Gu, Kate M. Candelario, Oleg Yegorov, and Duane A. Mitchell

Additional figures

**Fig. S1**


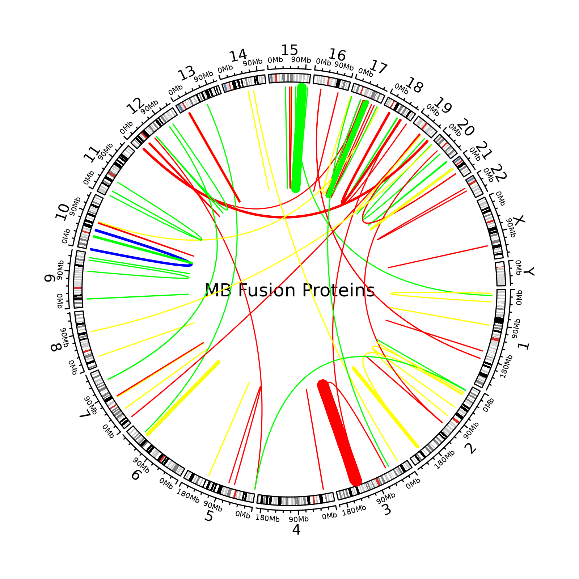


**Potentially targetable protein fusions in MB tumors.** Chord plot displaying fusion protein expression based on chromosome and subtypes for MB tumors. Patient tumor samples analyzed- n=170; 18 WNT (blue curve), 46 SHH (red), 41 Group 3 (yellow), and 65 Group 4(green). The thickness of the curve represents number of fusions from the same left protein and right protein.


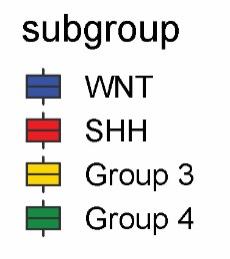

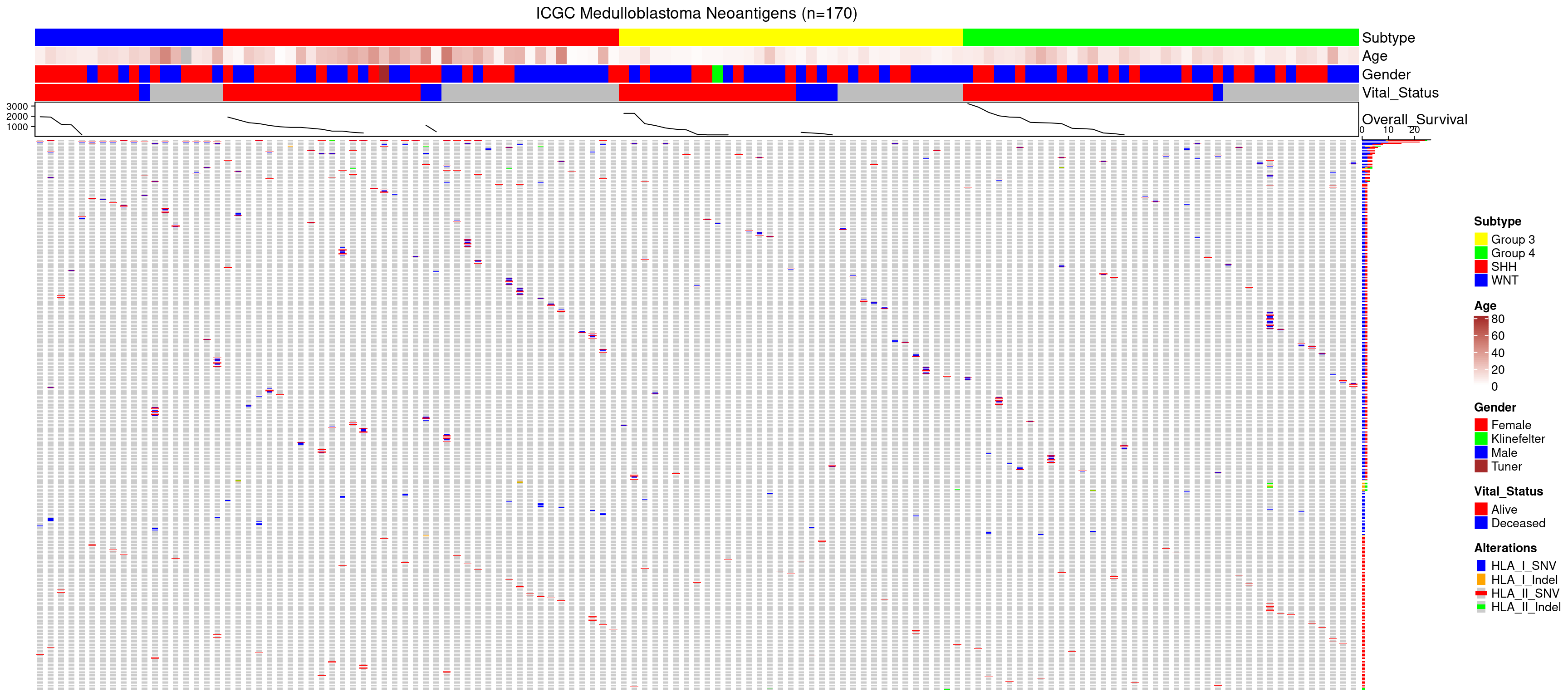


**Fig. S2**

**MHC Class I or Class II-restricted neoantigens in MB tumors. (A)** The HLA allotype distribution across the four MB subgroup patients. Most frequent HLA allotype are ordered from the left side. **(B)** Oncoprint representation of neoantigens predicted for all patient tumors across MB subtypes. Patient tumor samples analyzed- n=170; 18 WNT, 46 SHH, 41 Group 3, and 65 Group 4. Patients were ordered by the survival outcome from left to right as alive, deceased, or not available. Longest observed survival time to shortest was ordered from high to low. Most frequently recurring neoantigens were listed on the top.


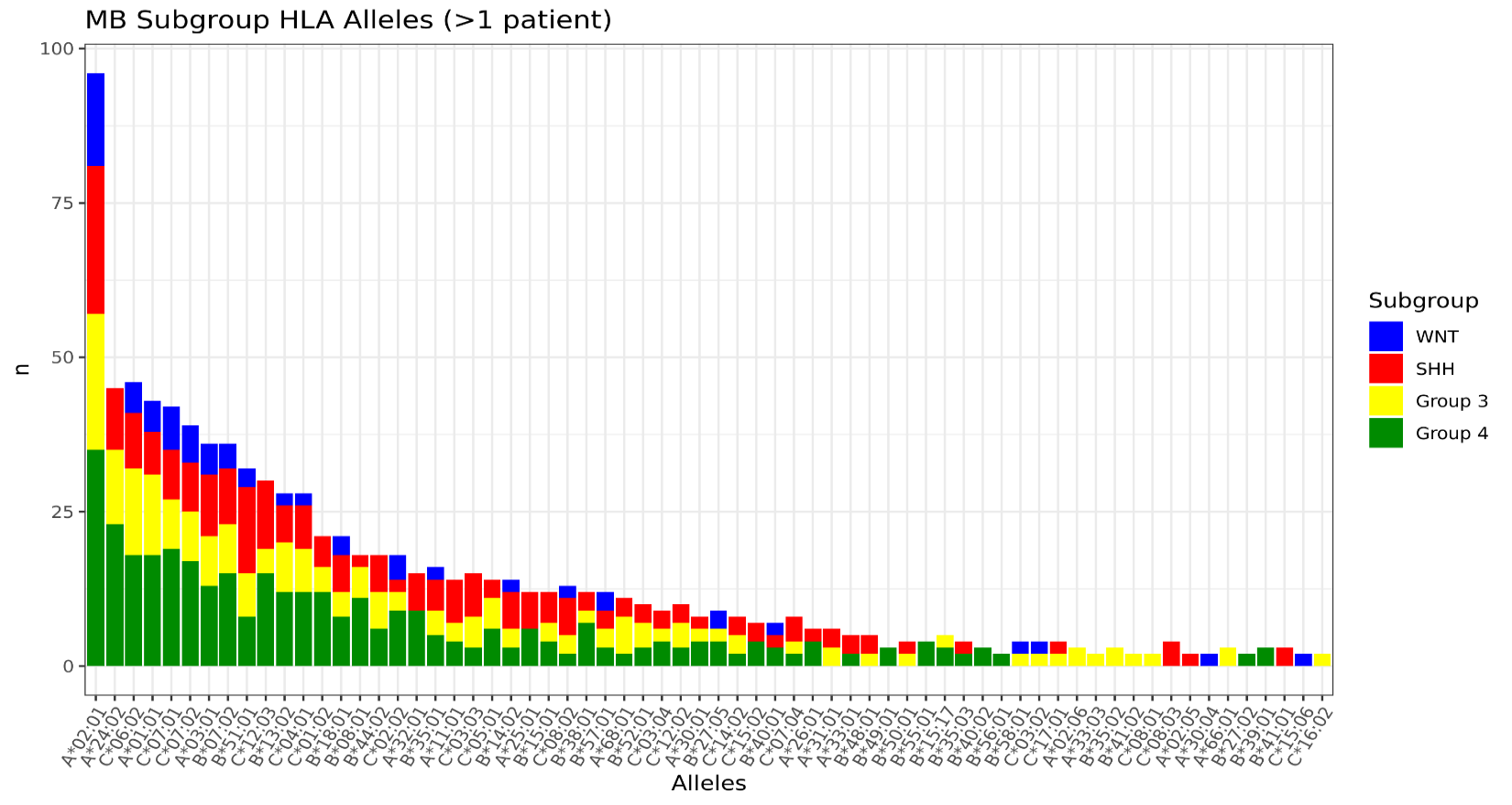


A

B

**Fig. S3**


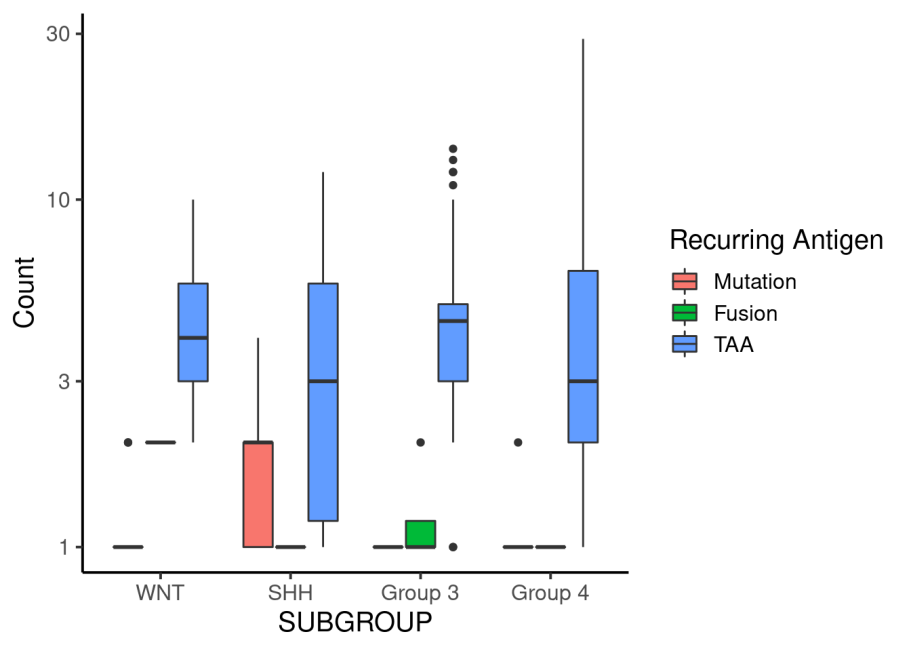


**Recurring antigens across MB molecular subtypes and correlation between antigen load and survival outcome. (A)** Quantitative data of Figure 4. Patient tumor samples analyzed- n=170; 18 WNT, 46 SHH, 41 Group 3, and 65 Group 4. Mutations (red bar), Fusions (green bar) and TAAs (blue bar) were plotted to represent recurring antigen count from each patient across four molecular subgroups. **(B, C)** Correlation between MHC-I and II restricted SNVs and progression-free survival in Group 4 medulloblastoma tumors. **(D,E)** Correlation between MHC-I and II restricted TAAs and overall survival in Group 3 medulloblastoma tumors. **(F,G)** Correlation between MHC-I and II restricted TAAs and progression-free survival in Group 3 medulloblastoma tumors.

Spearman correlation was performed between matched patients’ antigen and overall survival/progression-free survival. R > 0.3 indicates significant weak correlation. R > 0.6 indicates moderate correlation and R > 0.9 indicates strong correlation. Only significantly correlated relationship between antigens and survival was plotted.

_

A

B

F

D


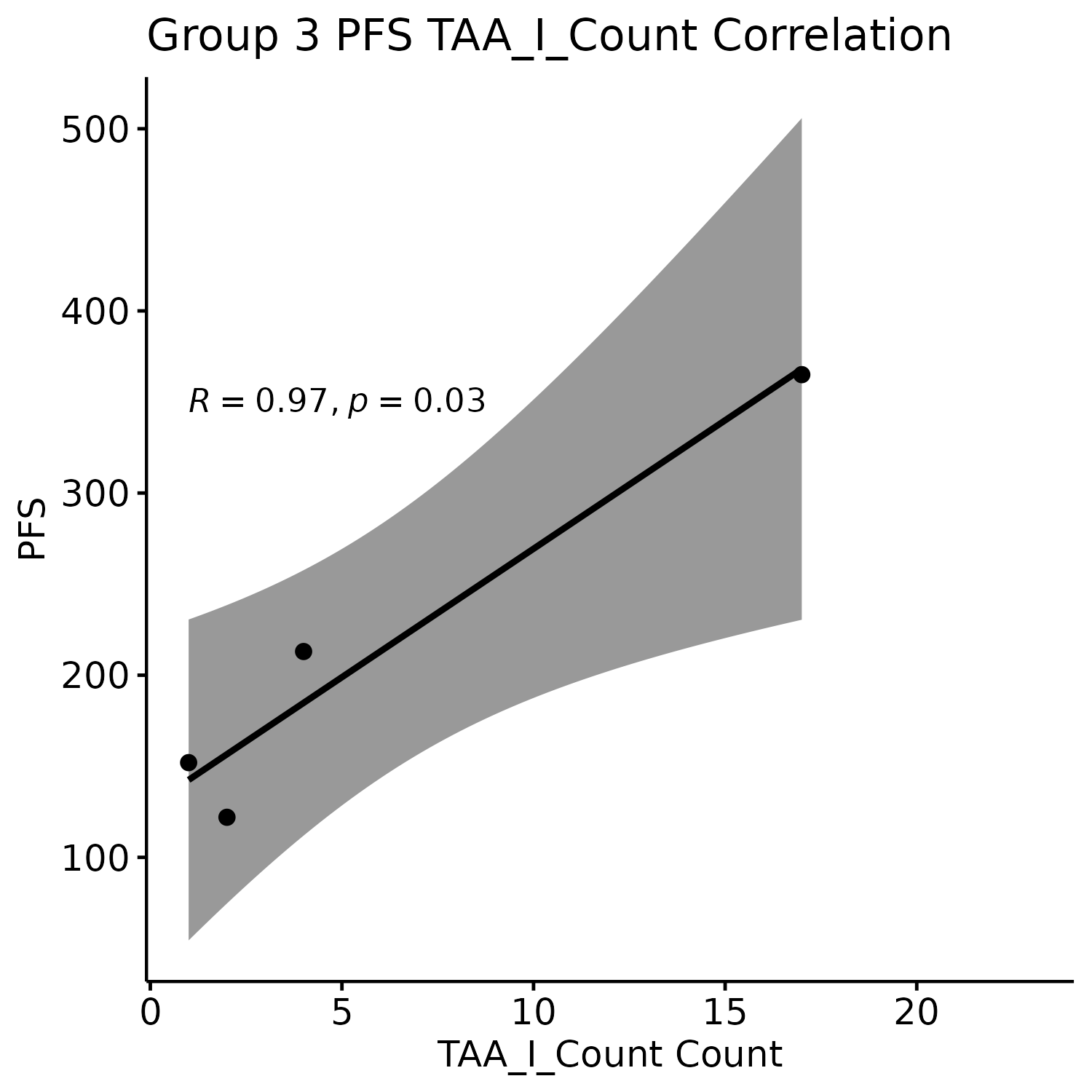

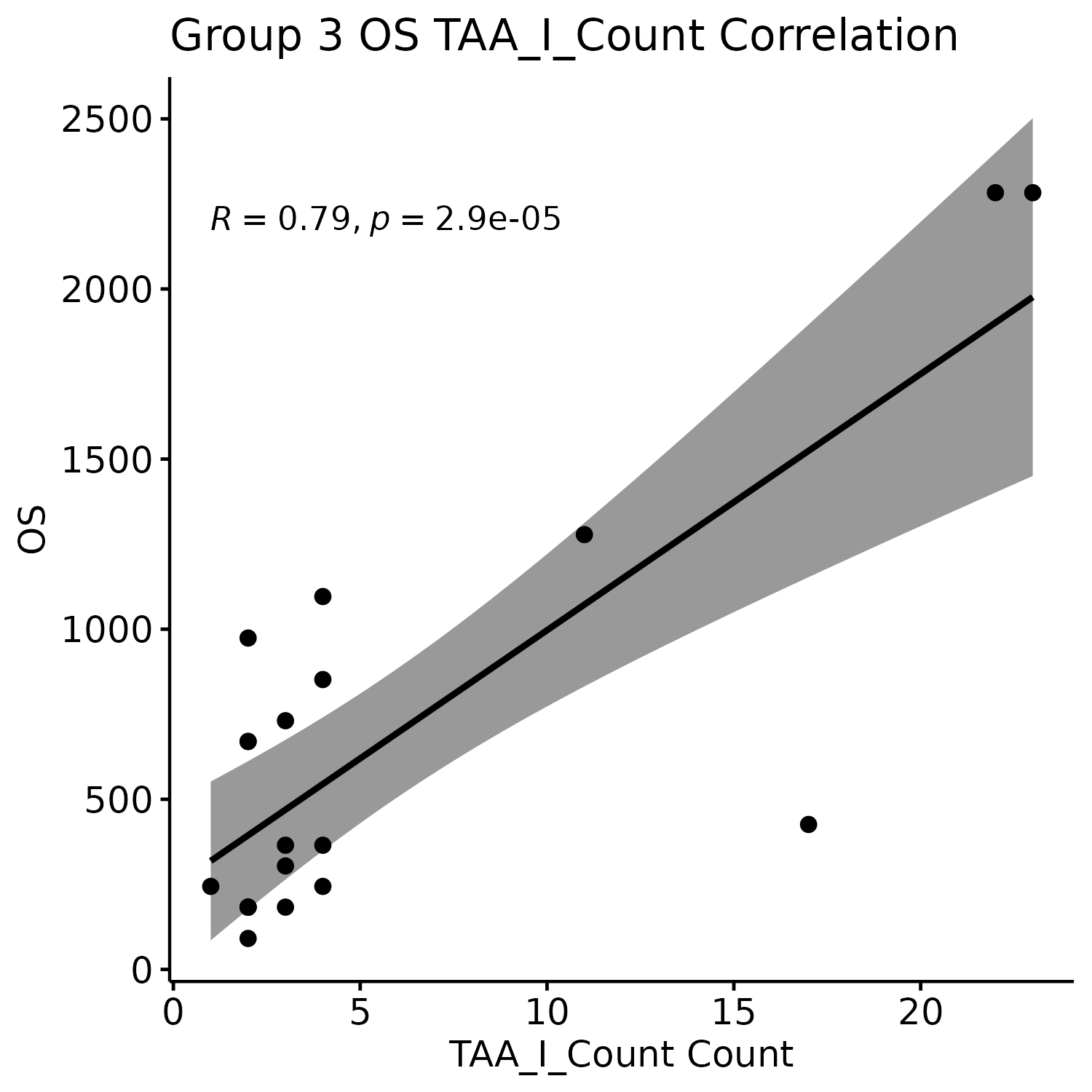

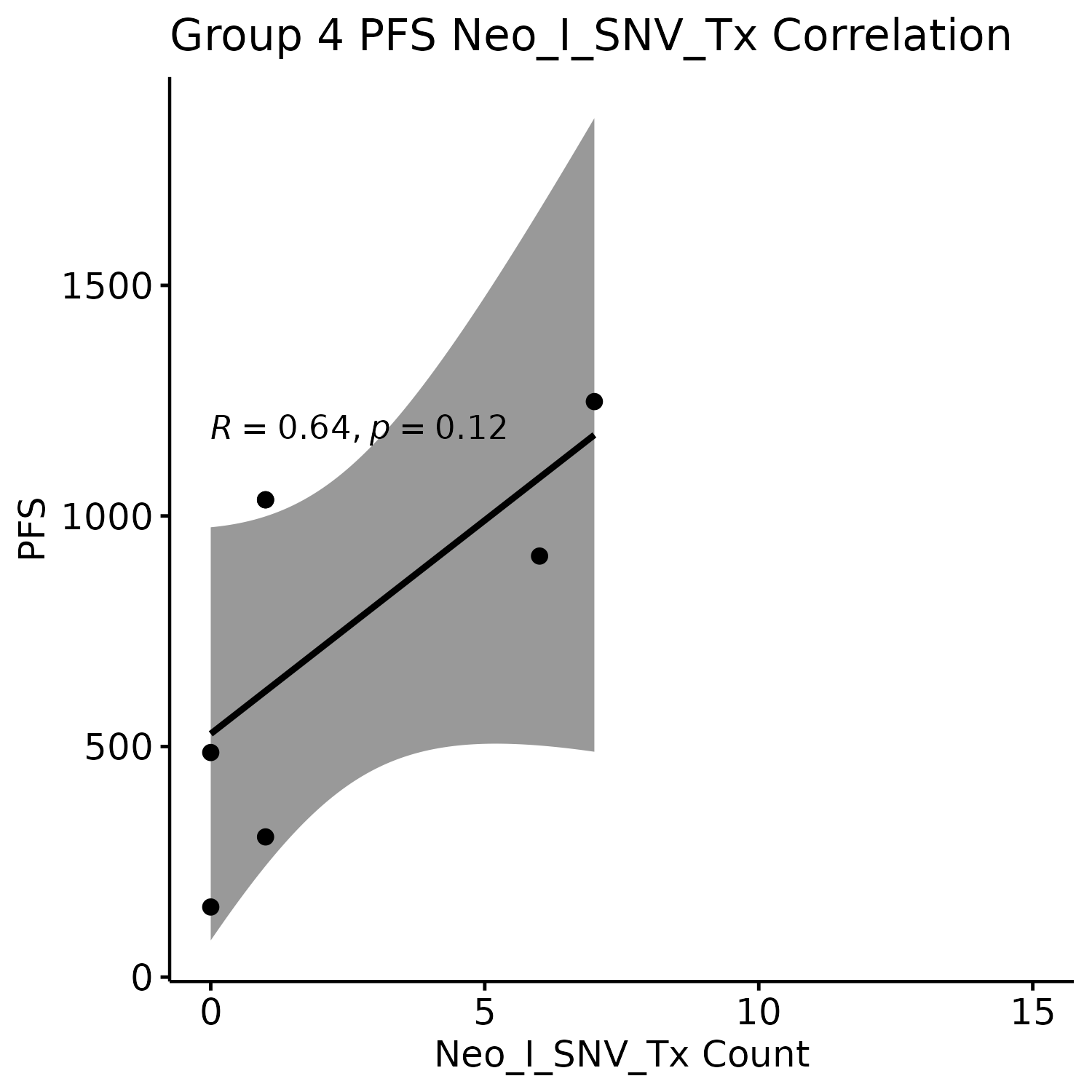


G

E

C


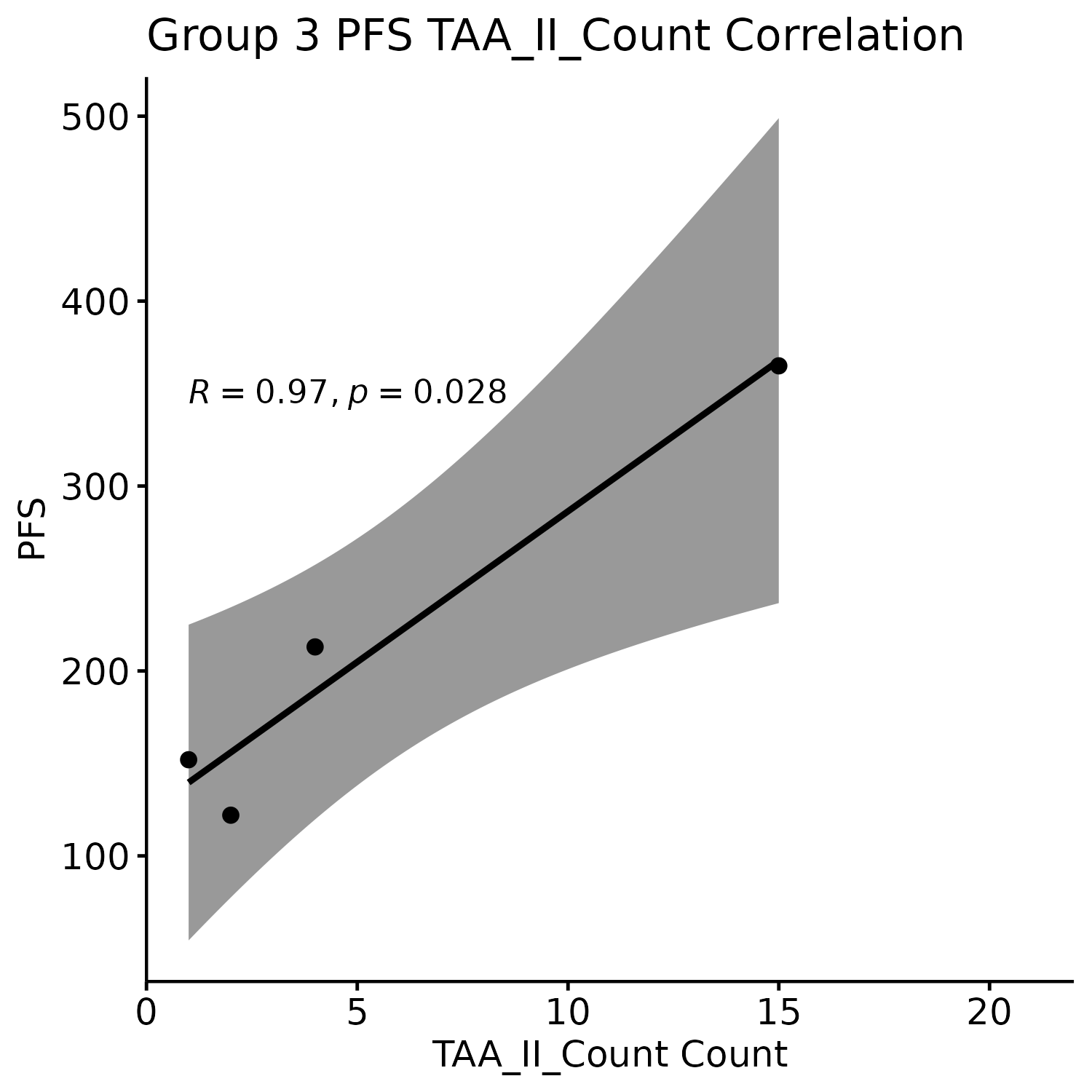

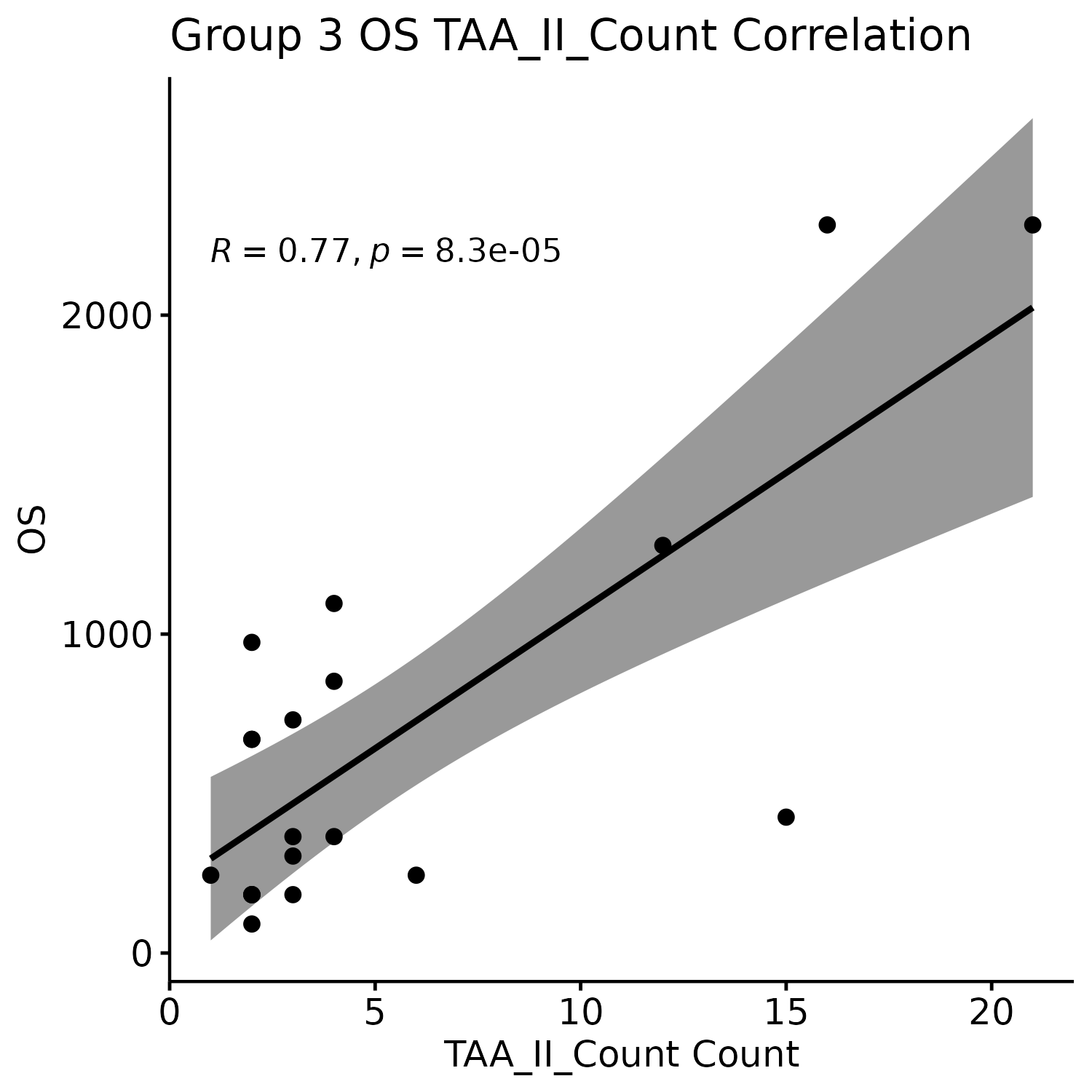

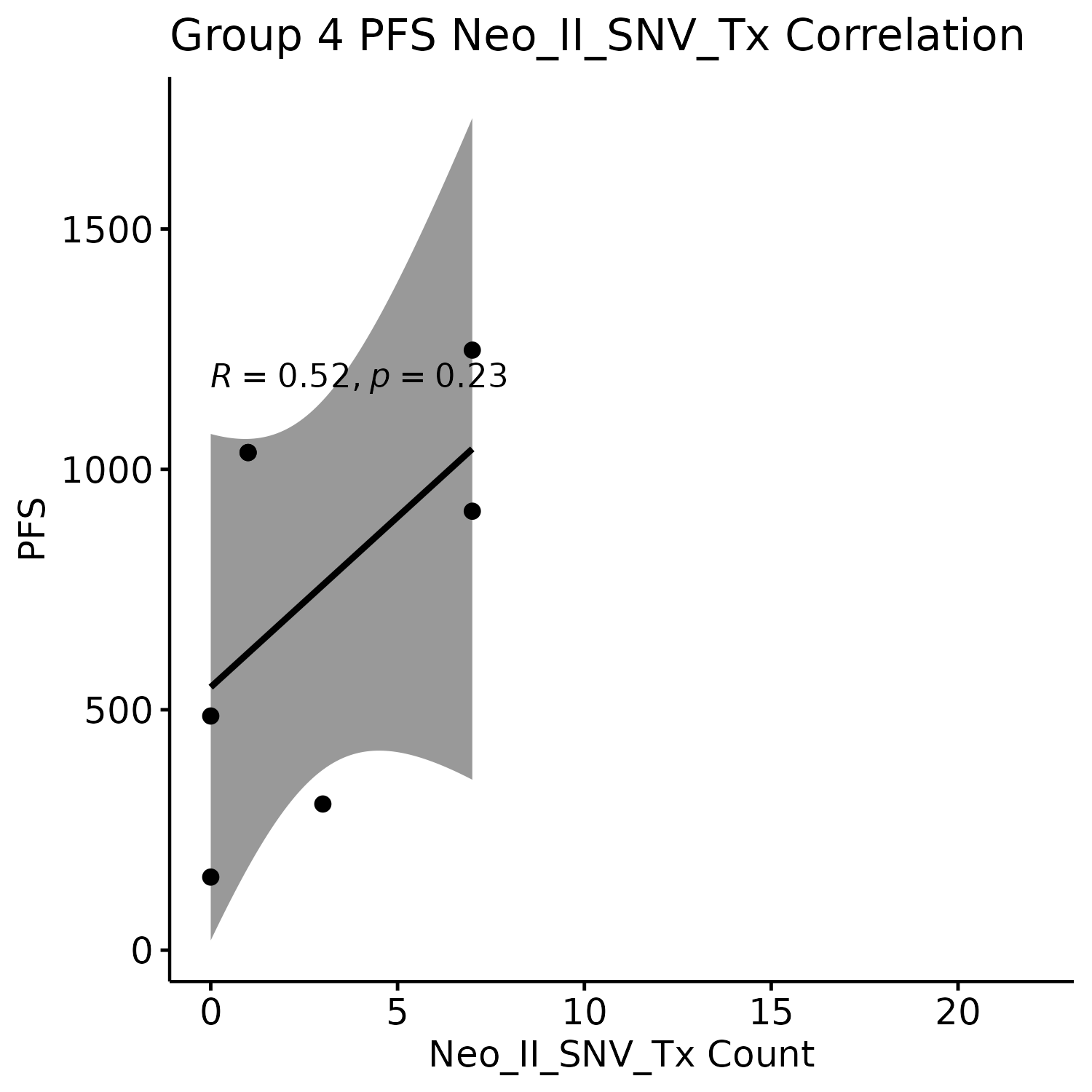


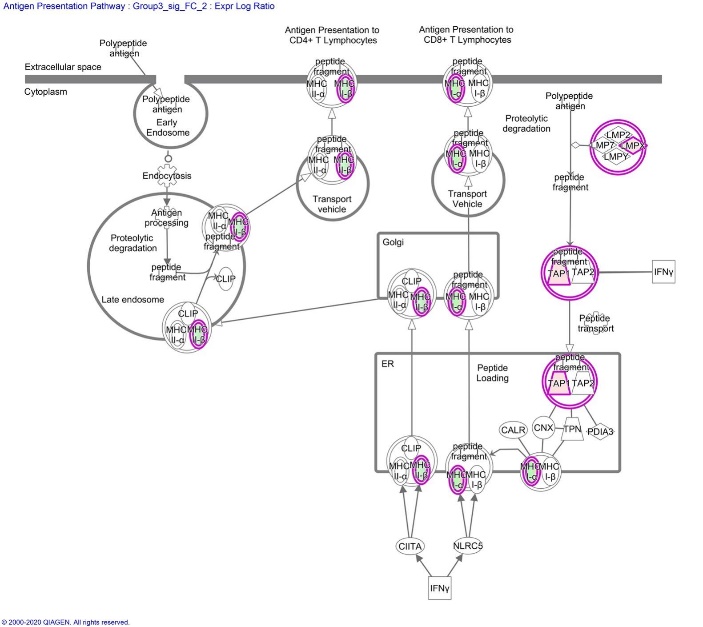


**Fig. S4**

**Antigen processing and presentation pathway in Group 3 MB tumors.** Downregulation of MHC-I and MHC-II antigen presentation pathway in the Group 3 subtype potentially impairing their ability to present antigens to the CD4 and CD8 T cells. The purple highlights show where in the antigen processing pathway important genes are being downregulated. Analysis was performed by using QIAGEN Ingenuity Pathway Analysis (IPA). Default settings was applied as Fisher’s exact test p < 0.05, and the ratio of Group 3 genes comparing to the rest subgroups over than 1-fold.
